# Cognitive rehabilitation using virtual reality in subjective cognitive decline and mild cognitive impairment: a systematic review

**DOI:** 10.3389/fpsyg.2025.1641693

**Published:** 2025-09-09

**Authors:** Maria Grazia Maggio, Raffaela Maione, Maria Cotelli, Piero Bonasera, Francesco Corallo, Giulia Pistorino, Antonina Luca, Angela Marra, Angelo Quartarone, Alessandra Nicoletti, Rocco Salvatore Calabrò

**Affiliations:** ^1^IRCCS Centro Neurolesi Bonino-Pulejo, Messina, Italy; ^2^Neuropsychology Unit, IRCCS Istituto Centro San Giovanni di Dio Fatebenefratelli, Brescia, Italy; ^3^Department of Medicine and Surgery, Università degli Studi di Messina, Messina, Italy; ^4^Department of Psychological Sciences and Techniques, Università degli Studi di Enna "Kore", Enna, Italy; ^5^Department of Medical, Surgical Sciences and Advanced Technologies "G.F. Ingrassia", University of Catania, Catania, Italy

**Keywords:** mild cognitive impairment, subjective cognitive decline, virtual reality-based cognitive training, cognitive rehabilitation, neurorehabilitation, early diagnosis

## Abstract

**Background:**

Mild Cognitive Impairment (MCI) and Subjective Cognitive Decline (SCD) are heterogeneous conditions that may indicate early dementia. Virtual Reality (VR) is emerging as a promising non-pharmacological tool for cognitive training. However, its effectiveness in these populations remains unclear. This systematic review examines the impact of VR-based cognitive interventions in individuals with SCD and MCI.

**Methods:**

A systematic review was conducted in accordance with PRISMA guidelines. Studies published between 2019 and 2025 investigating VR-based cognitive interventions in individuals diagnosed with SCD or MCI were identified through searches in PubMed, Scopus, Embase, and Web of Science. Eligible studies included randomized controlled trials (RCTs), experimental studies, and usability studies.

**Results:**

Nineteen studies met the inclusion criteria, including 14 RCTs, 2 usability studies, and 3 experimental studies. The majority of studies reported significant improvements in various cognitive domains, particularly memory, attention, and executive function, following VR-based interventions. Several studies also highlighted the positive impact of VR on user engagement and motivation, with high adherence and low dropout rates. However, there was considerable variability in intervention protocols, cognitive outcome measures, and participant characteristics. Most studies focused on individuals with MCI, while research on SCD populations remains limited and preliminary. Methodological quality varied, with some studies lacking adequate sample sizes or long-term follow-up.

**Conclusion:**

VR-based cognitive interventions appear to be a feasible and potentially effective approach for enhancing cognitive function in individuals with MCI, with emerging evidence also supporting their use in SCD. Despite encouraging results, further high-quality, large-scale trials are needed to validate these findings, standardize intervention protocols, and explore long-term benefits.

**Systematic review registration:**

CRD42025644894.

## 1 Introduction

Mild cognitive impairment (MCI) is considered an intermediate state between normal aging and dementia, characterized by cognitive deficits that do not yet significantly interfere with daily life (Sachdev et al., 2014). Among its subtypes, Amnestic MCI is particularly associated with Alzheimer’s disease and has a higher likelihood of progressing to dementia, whereas other subtypes may remain stable or even show improvement over time (Duff, 2024).

Subjective cognitive decline (SCD), characterized by self-reported cognitive difficulties despite normal performance on neuropsychological tests (Jessen et al., 2014) it is increasingly recognized as a potential precursor to MCI and dementia, particularly in individuals with biomarker evidence of AD pathology (Jessen et al., 2014; Jessen et al., 2020; Rabin et al., 2017; Reisberg et al., 2010).

Cognitive impairment, even in its early stages, is a growing public health concern, with MCI affecting approximately 15–20% of older adults and SCD reported by an even larger proportion (World Health Organization (WHO), 2021). SCD is particularly relevant as a potential warning sign of future cognitive decline. Studies estimate that 11.1% of individuals over 45 years old report SCD, with risk factors including aging, genetics, cardiovascular disease, diabetes, depression, and lifestyle factors (Bassett and Folstein, 1993; Bessi et al., 2018; GjØra et al., 2021; Liu et al., 2025; Rabin et al., 2015).

While SCD may remain stable or even improve, in some cases it represents the earliest detectable sign of underlying neurodegeneration, particularly in individuals with positive biomarkers for AD (Jessen et al., 2014). The transition from SCD to MCI is significant, marking the shift from subjective complaints to measurable cognitive deficits and highlighting the need for close monitoring of at-risk individuals (Rabin et al., 2017).

Although not all individuals with SCD or MCI will progress to dementia, these conditions increase the risk of neurodegeneration, making early identification and intervention crucial (Jack et al., 2011).

The early identification of SCD and MCI may offer a crucial window for intervention. Timely monitoring and targeted strategies can help delay or mitigate disease progression, emphasizing the importance of addressing cognitive concerns at the earliest stages (Jack et al., 2011).

Although individuals with SCD often maintain independence, many experiences daily challenges such as medication adherence, financial management, and household tasks (Tuokko and Smart, 2018). They also exhibit higher levels of distress, reduced social participation, and greater functional limitations, particularly in middle-aged adults (Wion et al., 2020).

Given the lack of disease-modifying treatments, there is a critical need for innovative non-pharmacological interventions aimed at maintaining cognitive function and delaying disease progression. In this context, VR-based cognitive interventions have gained attention for their ability to simulate real-world tasks, enhance cognitive engagement, and provide personalized, adaptive rehabilitation experiences (Yang et al., 2025). Virtual reality (VR) is an interactive computer-generated environment that simulates real-world settings, providing multisensory stimulation through visual, auditory, and motion-based feedback (Tieri et al., 2018). VR provides different degrees of “immersion” and “presence.” Immersion refers to the objective perceptual experience determined by the system’s features and the characteristics of the virtual task (i.e., the physical sensation of being in a virtual world). In contrast, presence is a subjective phenomenon, reflecting the user’s perceived involvement and emotional activation during the virtual experience (Tieri et al., 2018; Riva et al., 2020; Maggio et al., 2024). Based on the degree of immersion, VR interventions can be classified into three main categories: fully immersive VR, which involves the use of head-mounted displays (HMDs) or CAVE systems providing multisensory engagement; semi-immersive VR, which includes large screen-based simulations offering partial sensory involvement; and non-immersive VR, which refers to computer-based applications that offer minimal sensory integration and typically involve interaction through standard displays and interfaces (Tieri et al., 2018; Riva et al., 2020; Maggio et al., 2024).

Unlike traditional cognitive training, which often involves repetitive, abstract exercises, VR enables personalized, ecologically valid experiences that may mimic daily activities in a safe, controlled setting (Zhu et al., 2024). These features could enhance engagement, motivation, and adherence, key challenges in cognitive rehabilitation (Choi and Twamley, 2013).

Given the growing body of literature in the field, the present review seeks to address ongoing gaps focusing specifically on the feasibility, usability, and effectiveness of VR interventions in the earliest stages of cognitive decline, i.e., patients with SCD and MCI. Unlike previous meta-analyses, this systematic review provides an updated synthesis including the most recent studies published between 2019 and 2024 and highlights critical research gaps and future directions.

## 2 Methods

This systematic review investigated the use of VR interventions in populations with SCD, or MCI. The review protocol was registered on PROSPERO (Registration ID: CRD42025644894), ensuring methodological transparency and adherence to systematic review guidelines. The review followed the Preferred Reporting Items for Systematic Reviews and Meta-Analyses (PRISMA) 2020 guidelines (Page et al., 2021) to ensure a rigorous and reproducible approach to study selection and data synthesis. A comprehensive literature search was conducted in PubMed, Scopus, Embase, and Web of Science, covering studies published between December 2019 and August 2025. To ensure a systematic and reproducible approach, a combination of Medical Subject Headings (MeSH) terms and free-text keywords was used. The following key MeSH terms and keywords were used:

(“Virtual Reality”[MeSH] OR “Virtual Reality Training”) AND (“Neurorehabilitation”[MeSH] OR “Cognitive Rehabilitation” OR “Cognitive Training”) AND (“Mild Cognitive Impairment”[MeSH] OR “Cognitive Decline” OR “Subjective Cognitive Decline”) AND (“Cognition”[MeSH] OR “Executive Function” OR “Memory Disorders”[MeSH] OR “Attention”[MeSH] OR “Cognitive Dysfunction”[MeSH]) AND (“Quality of Life”[MeSH] OR “Activities of Daily Living”[MeSH]).

To ensure methodological rigor, the study selection process followed the PICO model (Population, Intervention, Comparison, Outcome):

- Population (P): Adults diagnosed with SCD, or MCI. Studies including mixed populations were considered only if data for SCD or MCI could be separately extracted.
- Intervention (I): VR-based interventions designed to improve cognitive, emotional, social, or functional outcomes. These included immersive VR (full 3D environments), semi-immersive VR (screen-based simulations), and non-immersive VR (computer-assisted cognitive training with VR elements). Only interventions involving VR, defined as interactive environments with a degree of immersion, were included. Serious Games without immersive VR components were excluded.
- Comparator (C): Studies comparing VR interventions with non-VR interventions (e.g., conventional cognitive training, standard physical therapy, traditional rehabilitation) or standard care were included. Studies without explicit comparators were considered if they provided pre- and post-intervention measures.
- Outcome (O): The primary outcomes assessed were cognitive improvements (e.g., changes in memory, attention, and executive functions) and emotional or functional enhancements (e.g., quality of life, social engagement, and daily functional abilities). Secondary outcomes included feasibility, and user satisfaction with VR interventions.

Studies were included if they investigated VR-based interventions aimed at enhancing cognitive, emotional, social, or functional outcomes in adults diagnosed with SCD or MCI. Only studies published in the last 5 years (2019–2024) were considered eligible. Regarding study design, randomized controlled trials (RCTs), non-randomized controlled trials, cohort studies, case–control studies, and cross-sectional studies were included. Only studies published in English were considered. No restrictions were applied based on open-access availability, as full texts were retrieved through institutional access.

Studies were excluded if they focused on paediatric populations, involved animal models, or did not include a VR intervention as the primary treatment. Additional exclusions applied to case reports, systematic reviews, meta-analyses, conference abstracts, study protocols, and proof-of-concept studies. Articles published outside the predefined timeline, those without full-text availability, or those lacking sufficient data were also removed.

The study selection process adhered to the PRISMA guidelines (Page et al., 2021) and was conducted using a blinded approach via Rayyan, a web-based tool designed for systematic reviews (Ouzzani et al., 2016). The use of Rayyan ensured that two independent reviewers (RM, RC) screened titles and abstracts separately in blinded manner, meaning they were unaware of each other’s decisions, thereby minimizing selection bias. During the first phase, studies were assessed based on predefined inclusion and exclusion criteria, and those clearly not meeting the eligibility requirements were excluded. In the second phase, full-text articles were reviewed with the blinding still in place. After the selection process was completed, blinding was removed, and a third reviewer (MGM) resolved any conflicts where consensus had not been reached. The level of agreement between the two primary reviewers regarding study inclusion was 80%, as estimated by Rayyan. Any discrepancies were discussed, and MGM acted as a tiebreaker when necessary. The entire study selection process was documented using a PRISMA flow diagram.

Following study selection, data extraction was performed independently by RM and RC using a structured extraction form in Microsoft Excel, with all entries cross-checked for accuracy. Extracted data included key study characteristics such as author information, publication year, country, and study design, along with participant details including sample size, mean age, sex distribution, and cognitive status (SCD, or MCI). Information about VR interventions was systematically collected, detailing the type of VR system used (immersive, semi-immersive, or non-immersive), as well as session duration, frequency, and intervention length. When applicable, comparator conditions, such as non-VR interventions or standard care, were also documented. Primary outcomes related to cognitive, emotional, social, and functional improvements were recorded alongside usability measures, including dropout rates and potential adverse effects such as cybersickness. Any discrepancies in data extraction were resolved through discussion, with MGM consulted when needed.

The risk of bias in randomized controlled studies was assessed using the Cochrane Risk of Bias (RoB2) tool, while the ROBINS-I tool was employed for non-randomized studies included in this review. Additionally, the overall quality of evidence for each outcome was evaluated using the GRADE (Grading of Recommendations, Assessment, Development, and Evaluation) framework (Guyatt et al., 2008) which considers factors such as risk of bias, inconsistency, indirectness, imprecision, and publication bias to provide a comprehensive evaluation of the strength of evidence.

As this systematic review did not involve meta-analysis due to the heterogeneity of study designs, intervention protocols, and outcome measures, data were synthesized using a qualitative narrative synthesis approach. The synthesis was structured according to the Synthesis Without Meta-analysis (SWiM) framework (Campbell et al., 2020) to ensure transparency and consistency in data interpretation. The findings were categorized based on key themes, including cognitive outcomes, emotional and psychological effects, functional improvements, and feasibility of VR interventions. Studies were grouped according to the type of VR intervention (immersive, semi-immersive, non-immersive), patient population (SCD, or MCI), and intervention setting (clinical, community, home-based). Results were summarized in tabular form, displaying key study characteristics, intervention details, and outcomes.

## 3 Results

**Figure 1:**
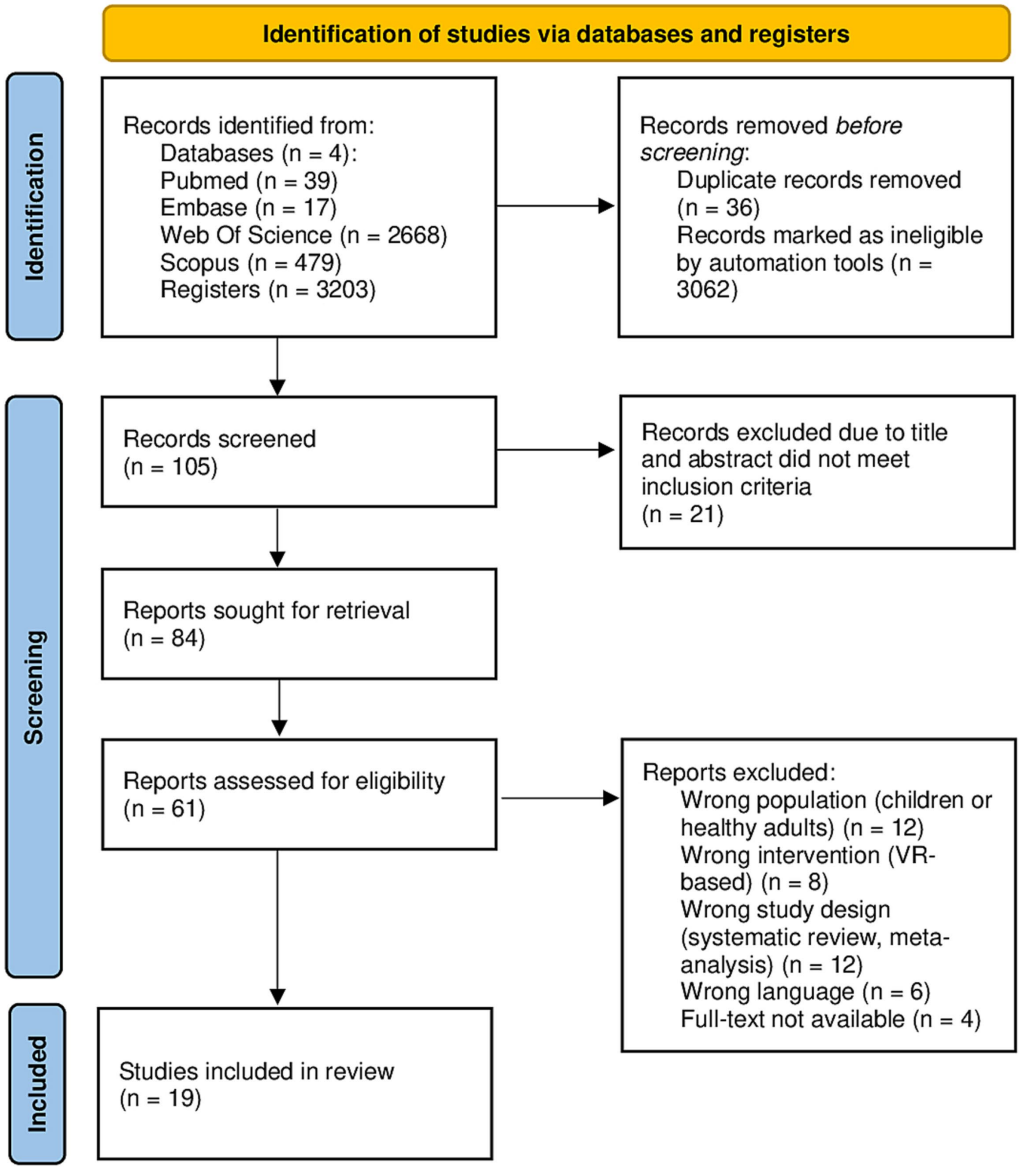
PRISMA 2020 flow diagram showing the identification, screening, eligibility, and inclusion process for studies assessing VR-based cognitive interventions in SCD and MCI populations.

The study selection process followed a systematic approach using predefined eligibility criteria before importing references into Rayyan for screening and deduplication. Initially, 3,203 articles were identified through database searches (PubMed, Scopus, Embase and Web of Science). After applying the eligibility criteria, which included language restrictions (English), publication timeframe (2019–2024), target population (SCD, and MCI), and intervention type (VR-based cognitive training), a total of 141 was deemed relevant for further screening. Using Rayyan, references were managed, screened, and deduplicated. Out of the 141, 84 studies were imported for screening, and 36 duplicates were identified and removed. Following the title and abstract screening, 23 studies were selected for full-text analysis. However, four articles were excluded due to the unavailability of full-text access, which resulted in missing critical information necessary for inclusion. This systematic review ultimately included 19 studies, consisting of 14 RCTs, 2 usability studies, and 3 experimental studies (Figure 1, Table 1, Supplementary Table 1) (Haddaway et al., 2022).

**Table 1:** Studies included in the analysis.

Author information	Type of study	Sample characteristic	Aim	Outcome measures	Main findings
Neumann et al. (2021)	Observational cross-sectional survey	*N* = 210; 105 participants with TBI: 60 men (39.0 ± 14.4) and 45 women (40.5 ± 12.9); 105 controls without TBI: 57 men (42.2 ± 15.1) and 48 women (39.3 ± 2.0)	The aim is to investigate gender differences in social inferencing deficits following traumatic brain injury (TBI) and to assess the likelihood of men and women experiencing impairment, while controlling for potential confounding factors.	Awareness of Social Inference Test (TASIT): Assesses affect recognition and theory of mind through three subtests: Emotion Evaluation Test (EET) (0–28), Social Inference-Minimal (SI-M) (0–60), Social Inference-Enriched (SI-E) (0–64); Stroop Color-Word Interference Test; Controlled Oral Word Association Test (COWAT); State–Trait Anxiety Inventory (STAI); Patient Health Questionnaire-9 (PHQ-9).	Comparing sex differences within the sample with TBI, women outperformed men on all 3 tasks. Although the initial findings indicated sex differences in emotion perception and mental state attribution after TBI, it was unclear from these group-based means how meaningful these differences were.
Teterina et al. (2023)	Observational cohort study	*N* = 276,812 patients aged 16–64 years from the National Ambulatory Care Reporting System (NACRS) and Discharge Abstract Database (DAD) datasets. The majority of the records (87%) were sourced from NACRS, with a slightly higher proportion of males (55.5%) compared to females (44.5%).	The aim of this article is to investigate the roles of sex (biological characteristics) and gender (social characteristics) in predicting outcomes after traumatic brain injury (TBI), specifically focusing on early mortality and discharge location. The study also aims to develop a method for measuring gender independently of sex, using a gender score to separate the effects of sex and gender on TBI outcomes, and to assess how these factors influence early mortality and discharge decisions after severe TBI.	No clinical tests were directly administered; instead, ICD-10-CA diagnostic codes, demographic data (age, sex, rurality, income), comorbidities (ADG score), injury severity, and discharge outcomes were used. A gender score was derived using logistic regression based on diagnostic patterns to estimate gender-related characteristics.	Sex (biological characteristics) significantly impacted early (30-day) mortality after severe traumatic brain injury (TBI), with males having a higher risk of early mortality compared to females, as indicated by a rate ratio of 1.54 (95% CI: 1.24–1.91). Gender (social characteristics) had a stronger influence than sex on discharge location after severe TBI. Individuals exhibiting more “woman-like” characteristics were less likely to be discharged to rehabilitation and more likely to be discharged home, with an odds ratio of 0.54 (95% CI: 0.32–0.88). The study developed a method to measure gender independently of sex, which allows for a better understanding of how both sex and gender contribute to TBI outcomes.
Wågberg et al. (2023)	Retrospective observational study	*N* = 595 patients who had suffered from mild traumatic brain injury (mTBI). Time Since Injury: Seven to eight years after the mild TBI. 40% of females and 29% of males reporting post-concussion symptoms. The study includes a broad age range, with a difference observed in the age group of 25–49 years, where women showed a higher level of disability compared to men. Some participants had experienced repeated mTBI, and the study examined the outcomes of those with multiple TBIs compared to those with a single mTBI.	The aim of the study is to evaluate post-traumatic brain injury (TBI) symptoms and disabilities seven to eight years after mild TBI (mTBI), with specific objectives to assess gender and age differences, and to examine whether repeated TBI leads to the deterioration of symptoms and function.	Rivermead Post-Concussion Symptoms Questionnaire (RPQ); Glasgow Outcome Scale Extended (GOSE).	Findings suggest the importance of considering gender and repeated TBI when planning rehabilitation and follow-up care for mTBI patients. 34% of patients reported symptoms, with more women (40%) affected than men (29%). Women had higher disability levels, with 31% not fully returning to daily life compared to 17% of men. The biggest difference was in the 25–49 age group. Patients with repeated TBIs had worse outcomes, with 31% not fully returning to daily life, compared to 21% for those with a single TBI.
Stafslien and Turkstra (2020)	Experimental study with a between-subjects design	*N* = 68 34 males (mean age = 20.91 years, SD = 0.75, range = 18–25) and 34 females (mean age = 19.53 years, SD = 1.58, range = 18–21), recruited from university students and other young adults in a midwestern city.	The aim of the study was to examine sex-based differences in expectations for social communication behaviors. It was expected that both men and women would have higher expectations for women’s social communication behavior compared to men’s, with women having the highest expectations for other women.	La Trobe Communication Questionnaire (LCQ).	Women were more critical than men when judging social behaviors, regardless of whether the individual being judged was male or female. The study highlights the importance of considering both the sex of the participant and the sex of the rater in social outcome research related to TBI. The findings suggest that sex-based differences in social evaluations may impact clinical settings, where female clinicians often assess male patients.
Adamson et al. (2021)	Observational cross-sectional study	*N* = 53 32 adults (16 females) with mild-to-severe traumatic brain injuries (TBI) and 21 neurologically healthy controls (11 females). Most TBI patients were from Santa Clara Valley Medical Center (28 civilians), with the rest from Veterans Affairs Palo Alto Health Care System (4 veterans). Healthy controls were recruited from Stanford University, local communities in Santa Clara County, and VAPAHCS staff (11 civilians, 10 veterans).	The aim of the study was to explore sex differences in cortical thickness and diffusion properties in patients with traumatic brain injury (TBI), and to compare these measures between TBI patients and neurologically healthy controls.	Ohio State University Traumatic Brain Injury Identification Method (OSU TBI-ID); Post Traumatic Stress Disorder PTSD Checklist (PCL-5); Trail Making Test B (TMTB); California Verbal Learning Test (CVLT); Repeatable Battery for the Assessment of Neuropsychological Status (RBANS); Wechsler Test of Adult Reading (WTAR); Mini-Mental State Exam (MMSE); select subtests from WAIS-IV (Digit Symbol, Digit Span); Semi-Structured Interview (assessed post-concussive symptoms based on ICD-10 criteria).	Findings contribute to understanding sex differences in brain structure and their implications for TBI rehabilitation. Patients with TBI showed greater cortical thinning in both hemispheres compared to healthy controls. Healthy females had significantly greater cortical (Adamson et al., 2021) compared to healthy males. However, this difference was less pronounced in the TBI group. There were no significant sex differences in diffusion properties (FA) among the participants. Moderate correlations were found between cortical thickness, diffusion properties, and cognitive performance, as assessed by the Trail Making Test B.
Levy et al. (2023)	Prospective cohort study	*N* = 108 68 adults with mild traumatic brain injury (mTBI) and 40 with general trauma (TC group), recruited from The Alfred Hospital and Royal Melbourne Hospital between December 2016 and January 2020. They were assessed 6–10 weeks post-injury.	the study was to examine self-reported cognitive symptoms in individuals with mild traumatic brain injury (mTBI) and trauma controls (TCs), and to explore psychological distress and gender as predictors of these symptoms.	Cognitive Complaint After Mild Closed Head Injury (CCAMCHI) scale; A-B Neuropsychological Assessment Schedule (ABNAS); Cognitive subscale of the Rivermead Post-Concussion Symptoms Ques tionnaire (RPQ); Inventory of Depressive Symptomatology (IDS; 30-item version); Beck Anxiety Inventory (BAI); PTSD Checklist for DSM-5 (PCL-5); Short-Form McGill Pain Question naire (SF-MPQ-2); Multidimensional Fatigue Inven tory (MFI); Weschler Test of Adult Reading (WTAR); Symbol Digit Modalities Test (SDMT); Digit Span subtest from the Weschler Adult Intelligence Scale, Fourth Edition (WAIS-IV); Rey Auditory Verbal Learning Test (RAVLT); Trail Making Test-Part B (TMT-B); Controlled Oral Word Association Test (COWAT)– letters F, A, and S. Measures were completed in the following order: SDMT, WTAR, RAVLT, DS, TMT B, COWAT-FAS, RPQ, ABNAS, CCAMCHI, MFI, IDS, BAI, PCL-5, and SF-MPQ-2.	Individuals with mTBI reported significantly higher subjective cognitive symptoms compared to trauma controls (TCs). Psychological distress and gender were significant predictors of subjective cognitive symptoms in the mTBI group. Specifically: Higher psychological distress was associated with greater cognitive symptoms. Females reported more severe cognitive symptoms than males. The findings suggest that mTBI-specific factors underlie the elevation in subjective cognitive symptoms, distinct from general post-concussion symptoms. These findings highlight the importance of considering gender and psychological distress when addressing cognitive symptoms in individuals with mTBI.

To ensure robustness, the risk of bias assessment integrated into the synthesis, highlighting whether higher-quality studies report consistent or conflicting findings.

**Figure 2:**
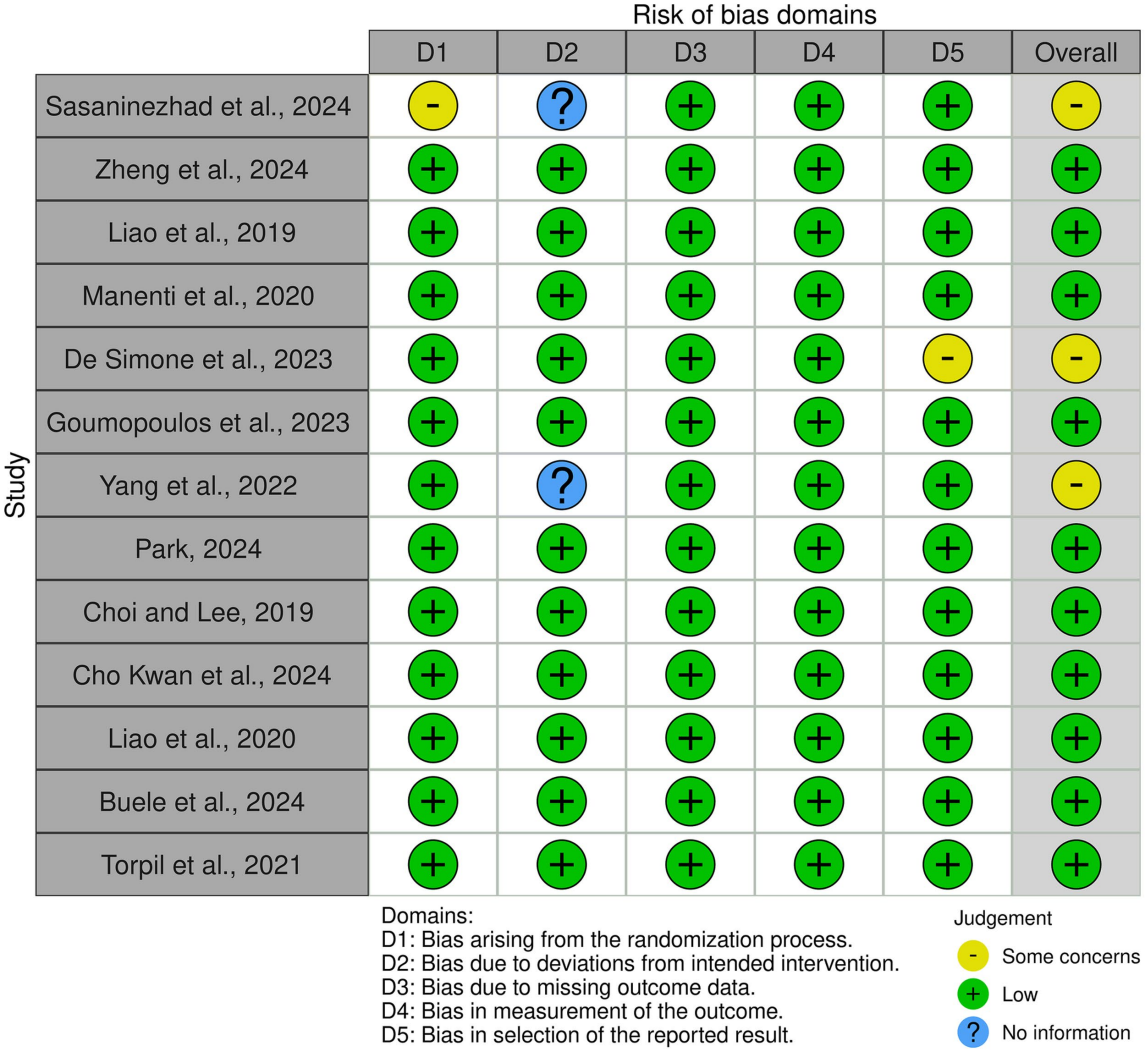
Summary of risk of bias assessments for randomized controlled trials using the Cochrane RoB2 tool. Green = low risk; Yellow = some concerns; Red = high risk.

The Cochrane Risk of Bias 2 (RoB 2) tool (Sterne et al., 2019) was applied to assess the quality of the randomized controlled trials (RCTs) included in this review. A total of 13 RCTs (Buele et al., 2024; Choi and Lee, 2019; De Simone et al., 2023; Goumopoulos et al., 2023; Kwan et al., 2024; Liao et al., 2019; Manenti et al., 2020; Park, 2024; Sasaninezhad et al., 2024; Torpil et al., 2021; Yang et al., 2022; Zheng et al., 2025; Liao et al., 2020) were identified and evaluated using RoB2. This tool examines bias across five key domains: (1) bias arising from the randomization process, (2) deviations from intended interventions, (3) missing outcome data, (4) measurement of outcomes, and (5) selection of reported results. Each study was classified as having low risk of bias (green), some concerns (yellow), or high risk of bias (red) (Figure 2).

The assessment revealed that most studies demonstrated a low risk of bias across all domains, supporting the reliability of their findings. However, some studies showed some concerns in specific areas. In particular, Sasaninezhad et al. (2024) exhibited some concerns regarding the randomization process, raising questions about allocation concealment or baseline imbalances. Additionally, the study did not provide clear information on deviations from the intended interventions, making it difficult to assess whether variations in protocol implementation may have influenced the results. De Simone et al. (2023) showed some concerns in the selection of reported results, suggesting a potential risk of selective reporting, which could impact the interpretation of the study’s findings. Yang et al. (2022) also had unclear information regarding deviations from intended interventions, limiting the ability to determine whether protocol adherence was consistent across participants.

Despite these concerns, the overall risk of bias was low for the majority of the included RCTs, suggesting that the findings of these studies provide robust evidence for the efficacy and feasibility of VR-based interventions in individuals with SCD, or MCI. Nonetheless, caution should be taken when interpreting results from studies with identified methodological limitations.

**Figure 3:**
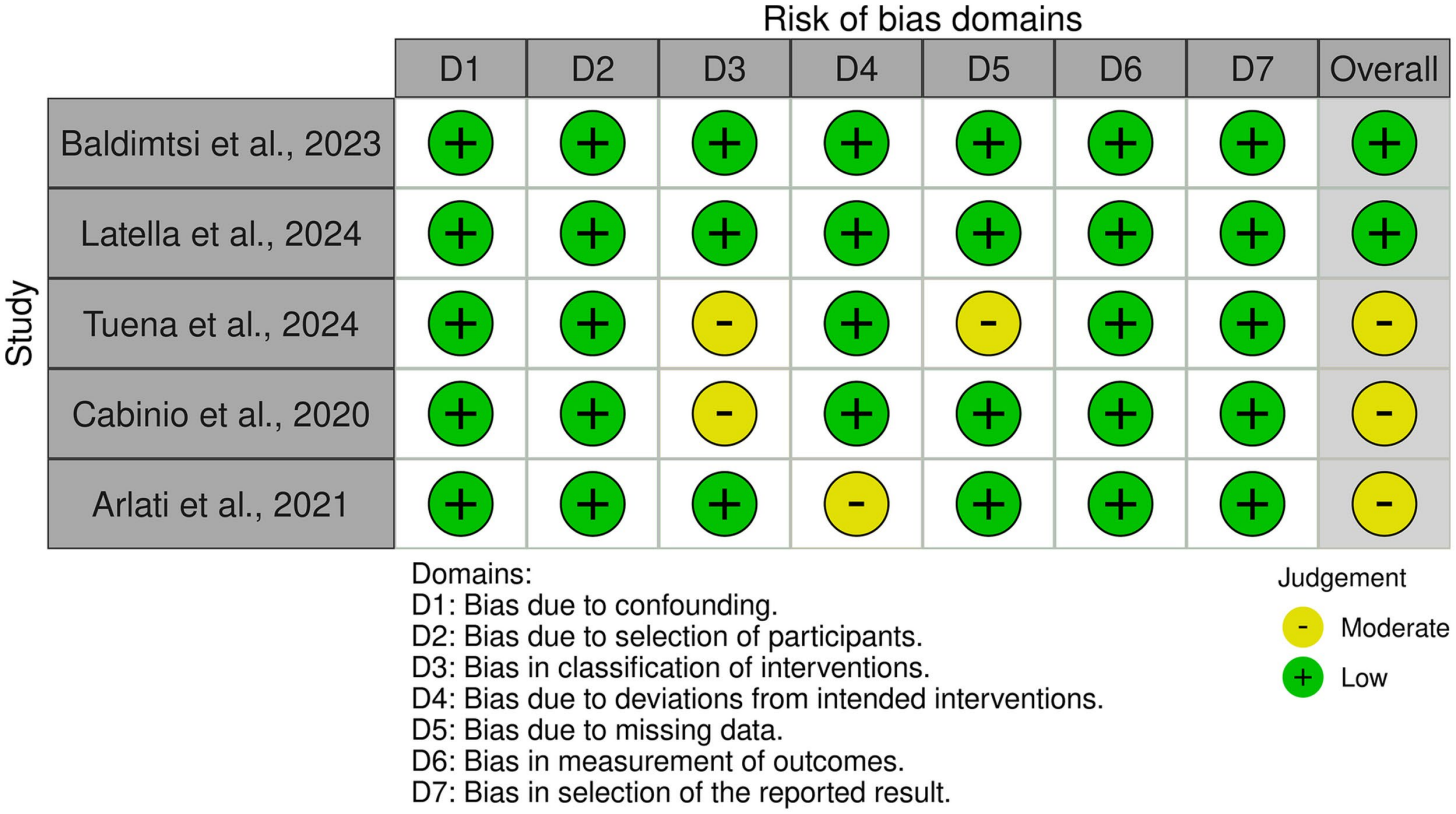
Risk of bias assessment for non-randomized studies using the ROBINS-I tool. Most studies showed low to moderate risk across evaluated domains.

For non-randomized studies, including cohort and case–control studies, the Risk of Bias in Non-randomized Studies of Interventions (ROBINS-I) tool (Sterne et al., 2016) was employed to assess methodological quality across seven key domains: (1) bias due to confounding, (2) bias due to selection of participants, (3) bias in classification of interventions, (4) bias due to deviations from intended interventions, (5) bias due to missing data, (6) bias in measurement of outcomes, and (7) bias in selection of the reported result. Each study was classified as having low risk (green) or moderate risk (yellow) of bias in the respective domains (Figure 3). A total of five non-randomized studies (Arlati et al., 2021; Baldimtsi et al., 2023; Cabinio et al., 2020; Latella et al., 2024; Tuena et al., 2024) were assessed using the ROBINS-I framework. The majority of studies exhibited low risk of bias across most domains, supporting the reliability of their findings. However, some concerns were noted in certain areas. Tuena et al. (2024) exhibited moderate risk of bias due to missing outcome data and selection of reported results, suggesting potential limitations in data completeness and transparency in reporting findings. Cabinio et al. (2020) also presented moderate risk in missing outcome data, which may impact the reliability of the reported conclusions. Arlati et al. (2021) had moderate concerns regarding deviations from intended interventions, indicating possible inconsistencies in the application of the intervention that could introduce variability in the study outcomes.

Despite these minor concerns, the overall risk of bias was predominantly low, reinforcing the credibility of the included non-randomized studies. However, the presence of moderate bias in some domains suggests that results should be interpreted with caution, particularly concerning intervention classification and data completeness.

In addition to evaluating the risk of bias using the RoB 2 tool for RCTs and the ROBINS-I tool for non-randomized studies, we also assessed the overall quality of evidence using the GRADE (Grading of Recommendations, Assessment, Development, and Evaluation) framework. The GRADE approach considers five key factors: risk of bias, inconsistency, indirectness, imprecision, and publication bias.

For each outcome, we assigned a quality rating (high, moderate, or low) based on these criteria. The final assessment is summarized in Table 2.

**Table 2:** GRADE (grading of recommendations, assessment, development, and evaluation) evaluation of the studies.

Outcome	Study design	Risk of bias	Inconsistency	Indirectness	Imprecision	Publication bias	Overall quality of evidence
MCI	RCT, intervention study, experimental study and feasibility and usability study	Low	Serious	Serious	Low	Low	Low
SCD	Usability and acceptance study	Low	Not serious	Serious	Serious	Low	Low–Moderate

RCT, Randomized Controlled Trial; MCI, Mild Cognitive Impairment; SCD, Subjective Cognitive Decline.

The GRADE assessment revealed variability in the overall quality of evidence across different outcomes. The quality of evidence for MCI was low, reflecting a combination of well-conducted RCTs, intervention study, experimental study and feasibility and usability study. Evidence for using of VR in SCD showed moderate overall value, except for indirectness. The strength of evidence was evaluated using standardized GRADE criteria, considering factors such as study design, sample size, risk of bias, and the reproducibility of results (see Table 2).

The studies investigated VR-based interventions in populations with SCD and MCI, assessing their effects on cognitive function, daily activities, emotional well-being, usability, and adherence. Among the studies analysed, the majority were randomized controlled trials (RCTs) (*n* = 15), while five were non-RCTs, including usability, feasibility, and intervention studies (Arlati et al., 2021; Baldimtsi et al., 2023; Cabinio et al., 2020; Latella et al., 2024; Tuena et al., 2024). The total sample size across the RCTs was 1,175 participants, with individual studies ranging from 21 to 293 participants. The non-RCT studies included an additional 384 participants, bringing the total sample size to 1,559 individuals. Most studies focused on MCI, with only one study (Arlati et al., 2021) also including individuals with SCD. The majority utilized immersive VR systems, such as head-mounted displays (HMDs) and motion controllers, including Oculus Quest/Oculus Go (Buele et al., 2024; De Simone et al., 2023; Yang et al., 2022; Zheng et al., 2025; Baldimtsi et al., 2023; Tuena et al., 2024), and HTC Vive (Kwan et al., 2024; Arlati et al., 2021). Some studies used semi-immersive VR, relying on screen-based simulations (Park, 2024; Sasaninezhad et al., 2024) or non-immersive VR, such as VR cognitive training (Latella et al., 2024) and Microsoft Kinect (Liao et al., 2019; Torpil et al., 2021). One study combined VR with neurofeedback using functional near-infrared spectroscopy (fNIRS) (Park, 2024). Cabinio et al. (2020) used a touchscreen interface to assess cognitive abilities in a virtual home environment. Training durations varied from a single session (Cabinio et al., 2020) to 5 months (Latella et al., 2024), with most studies lasting between 4 to 12 weeks. The number of participants in each study was generally balanced across intervention and control groups, ensuring methodological rigor.

The majority of the studies (17 out of 18) focused on individuals with MCI, assessing the effects of VR-based cognitive and physical training on executive function, memory, and daily functioning (Buele et al., 2024; Choi and Lee, 2019; De Simone et al., 2023; Goumopoulos et al., 2023; Kwan et al., 2024; Liao et al., 2019; Manenti et al., 2020; Park, 2024; Sasaninezhad et al., 2024; Torpil et al., 2021; Yang et al., 2022; Zheng et al., 2025; Liao et al., 2020). In contrast, only one study (Arlati et al., 2021) investigated the application of VR interventions in individuals with SCD.

Several RCTs have demonstrated the effectiveness of VR-based training in enhancing cognitive and functional outcomes in MCI. Zheng et al. (2025) reported significant improvements in cognition and instrumental ADL following VR interventions. Moreover, Liao et al. (2020, 2019) found that cognitive-motor training improved dual-task performance, balance, and cognitive flexibility. Similarly, Yang et al. (2022) showed that VR-based dual-task exercises enhanced both cognitive and physical health. Expanding on these findings, Kwan et al. (2024) and Park (2024) further explored VR-based dual-task interventions, emphasizing that simultaneous cognitive and motor training strengthens cognitive-motor interactions and promotes neuroplasticity. In addition to its role in dual-task training, VR has been compared to traditional cognitive exercises, showing comparable or even superior efficacy. De Simone et al. (2023) reported that VR-based executive function training led to superior memory and problem-solving improvements in Parkinson’s disease with MCI compared to placebo-controlled interventions. Similarly, Goumopoulos et al. (2023) found that a VR-based cognitive training platform (COGNIPLAT) was more effective than usual care in enhancing attention, processing speed, and executive function.

Beyond cognitive benefits, several studies investigated the acceptability, and usability of VR interventions. De Luca et al. (2024) found that VR training was highly usable, with strong engagement levels. Moreover, Choi and Lee (2019) and Tuena et al. (2024) emphasized that VR usability depends on individual cognitive profiles, with older adults benefiting from task customization to improve engagement.

Lastly, Cabinio et al. (2020) and Liao et al. (2019) explored the use of VR navigation aids to enhance spatial memory recall. Their findings suggested that VR-based spatial training may improve functional abilities in individuals with MCI, offering a potential intervention for early cognitive decline.

Notably, only one study focused on SCD. Arlati et al. (2021) and Baldimtsi et al. (2023) investigated the feasibility and early effects of VR-based cognitive training, assessing usability and engagement. Their results indicated that immersive VR cognitive stimulation improved spatial navigation and executive function. However, some participants required additional guidance to navigate more complex VR tasks, highlighting the need for tailored support in this population.

Overall, VR interventions showed strong potential for cognitive training, particularly in executive function, memory, and cognitive-motor integration. Studies highlighted the importance of personalization, usability optimization, and long-term follow-ups to maximize clinical impact. While VR-based cognitive training shows promise as an early intervention for SCD, further research is needed to determine whether it can effectively prevent or slow cognitive decline in this population.

Given the heterogeneity of the included interventions, it is also important to consider how the degree of VR immersion and the specific cognitive content may have influenced the observed outcomes. Across the included studies, interventions employing fully immersive VR systems (e.g., head-mounted displays) tended to report greater improvements in cognitive outcomes compared to semi-immersive or non-immersive systems, although findings were not entirely consistent (Buele et al., 2024; Choi and Lee, 2019; De Simone et al., 2023; Goumopoulos et al., 2023; Kwan et al., 2024; Manenti et al., 2020; Park, 2024; Sasaninezhad et al., 2024; Torpil et al., 2021; Yang et al., 2022; Zheng et al., 2025; Liao et al., 2020; Arlati et al., 2021; Baldimtsi et al., 2023; Cabinio et al., 2020; Latella et al., 2024; Tuena et al., 2024). Importantly, the therapeutic content appeared to play a decisive role: studies targeting memory and executive functions showed more robust benefits than those focused on general cognitive stimulation or attention alone. These observations suggest that both the degree of immersion and the specificity of the cognitive tasks may modulate the effectiveness of VR-based interventions.

## 4 Discussion

**Figure 4:**
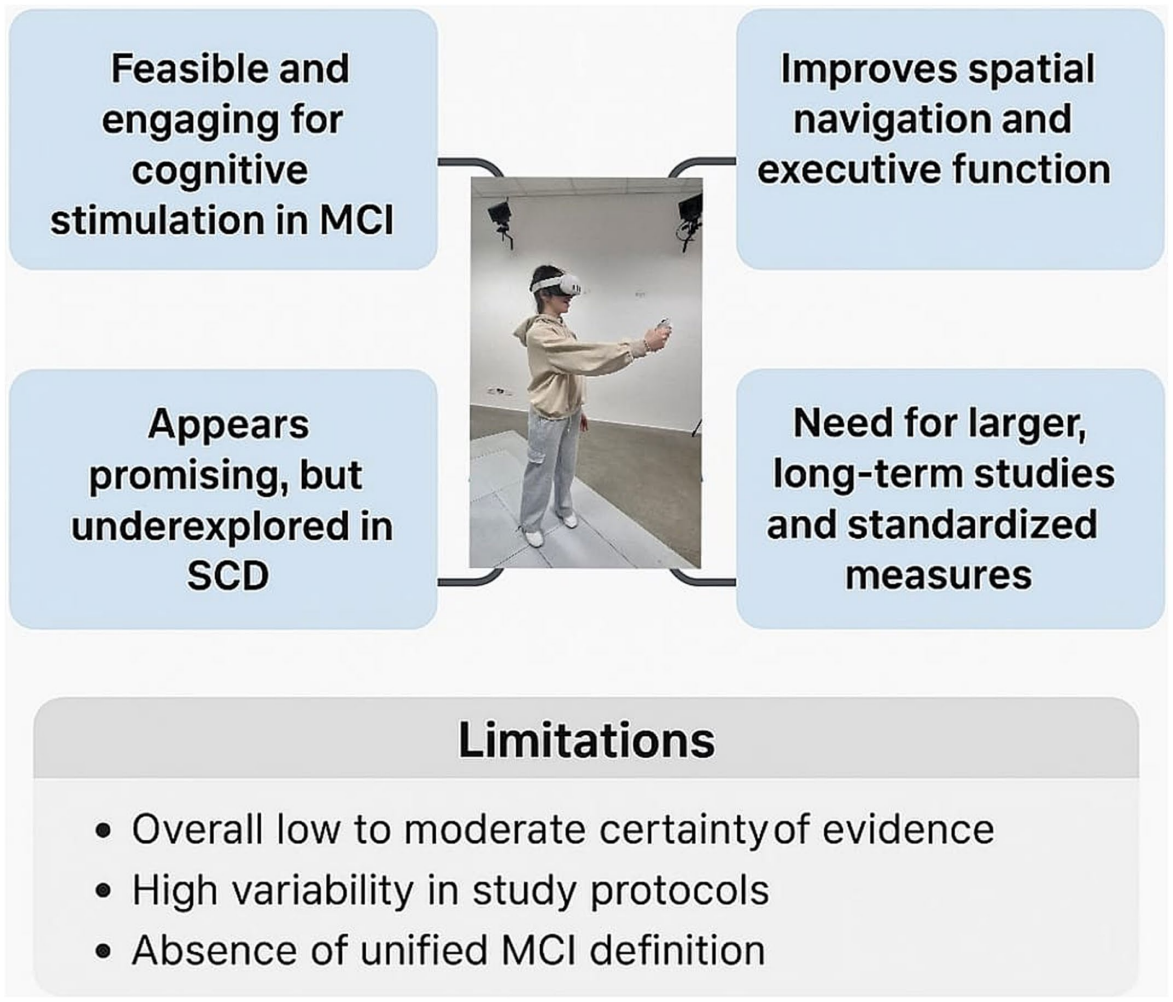
Summary of key findings and limitations of VR-based cognitive interventions in MCI and SCD.

The present systematic review underscores the potential role of VR-based interventions in individuals experiencing cognitive decline (Figure 4).

Compared to previous systematic reviews and meta-analyses (Kim et al., 2019; Voinescu et al., 2024) focusing primarily on MCI and dementia, this review expands the scope by also including SCD as a prodromal phase and highlights feasibility and usability as critical outcomes for the future application of VR interventions. The findings suggested that VR interventions can enhance cognitive functions, particularly memory, attention, and executive functions, while also promoting engagement and adherence through their immersive and interactive nature. Furthermore, VR has shown potential in addressing emotional and psychological well-being, reducing anxiety, and improving mood, which are critical aspects in the early stages of cognitive decline (Buele et al., 2024; Choi and Lee, 2019; De Simone et al., 2023; Goumopoulos et al., 2023; Kwan et al., 2024; Liao et al., 2019; Manenti et al., 2020; Park, 2024; Sasaninezhad et al., 2024; Torpil et al., 2021; Yang et al., 2022; Zheng et al., 2025; Liao et al., 2020). Notably, while VR provides an innovative and engaging medium, its therapeutic efficacy appears to be primarily driven by the specific cognitive content delivered within the VR environment, such as memory training, executive function exercises, and spatial navigation tasks. Therefore, VR could be conceptualized as a therapeutic delivery platform rather than an intervention in itself. Moreover, most VR-based interventions have been tested in individuals with MCI (Buele et al., 2024; Choi and Lee, 2019; De Simone et al., 2023; Goumopoulos et al., 2023; Kwan et al., 2024; Liao et al., 2019; Manenti et al., 2020; Park, 2024; Sasaninezhad et al., 2024; Torpil et al., 2021; Yang et al., 2022; Zheng et al., 2025; Liao et al., 2020) with only one study explicitly focused on SCD (Arlati et al., 2021), highlighting a gap in research that warrants further exploration. This is consistent with broader literature trends, where VR has been primarily investigated in individuals with MCI. In contrast, its application in the very earliest stage, SCD, remains largely unexplored. The concentration of studies on MCI suggests that researchers view this stage as particularly amenable to cognitive interventions, as individuals retain sufficient cognitive resources to engage and benefit from digital and immersive rehabilitation tools. The concentration of studies in MCI suggests that researchers consider this stage particularly responsive to cognitive interventions, as individuals retain sufficient cognitive resources to engage and benefit from digital and immersive rehabilitation tools. In patients with MCI, the included studies demonstrated that VR-based cognitive and cognitive-motor interventions produced significant improvements in executive function, memory, and dual-task performance (Buele et al., 2024; Choi and Lee, 2019; De Simone et al., 2023; Goumopoulos et al., 2023; Kwan et al., 2024; Liao et al., 2019; Manenti et al., 2020; Park, 2024; Sasaninezhad et al., 2024; Torpil et al., 2021; Yang et al., 2022; Zheng et al., 2025; Liao et al., 2020). These findings are consistent with the existing literature on VR applications in neurorehabilitation, where similar results have been reported in several neurological conditions (Maggio et al., 2023; Morone et al., 2014). In fact, it has been demonstrated that VR-based cognitive-motor training improved executive function and motor performance in stroke survivors, suggesting that the integration of physical activity with cognitive exercises in a VR environment may promote neuroplasticity. While both physical activity and cognitive exercises have established benefits, VR uniquely supports their integration within ecologically valid, adaptive environments. This multimodal stimulation may better approximate real-world cognitive-motor demands, enhancing the transferability of skills to daily life. Similarly, VR-based cognitive-motor exercises could improve executive control and divided attention, further reinforcing the interconnected nature of cognition and movement in neurodegenerative conditions (Choi and Lee, 2019; De Simone et al., 2023; Goumopoulos et al., 2023; Kwan et al., 2024; Liao et al., 2019; Manenti et al., 2020; Park, 2024; Sasaninezhad et al., 2024; Torpil et al., 2021; Zheng et al., 2025; Specht et al., 2023; Yang et al., 2022). Regarding the degree of immersion, fully immersive VR systems (e.g., head-mounted displays) have been associated with greater improvements in cognitive outcomes compared to semi-immersive or non-immersive systems. However, immersion alone is not necessarily the key therapeutic factor. Instead, its clinical relevance lies in the ability to foster embodied cognition, enhance attentional engagement, and simulate real-life complexity in a controlled and adaptable environment (Tieri et al., 2018; Maggio et al., 2022). This supports more effective generalization of skills beyond the training context. Nonetheless, across the reviewed studies, therapeutic content, particularly programs targeting memory and executive functions, emerged as a more critical determinant of outcomes than immersion per se. Thus, VR-based interventions appear to be most effective when they combine meaningful cognitive content with interactive, ecologically valid environments. However, therapeutic content emerged as a more critical determinant of outcomes. Indeed, interventions focused on memory and executive functions yielded the most consistent cognitive benefits across studies, independent of the level of immersion. Therefore, VR-based interventions appear to be particularly effective when combining cognitive and physical training. Some studies included in our review demonstrated that MCI participants engaged in VR-based dual-task training showed better cognitive and motor outcomes than those undergoing traditional interventions (Liao et al., 2019; Yang et al., 2022). Moreover, alterations in gait parameters have been consistently observed in individuals with dementia, including reduced gait speed, increased stride time variability, and impaired gait stability, especially under dual-task conditions (Chiaramonte and Cioni, 2021). These motor deficits are closely linked to cognitive dysfunction and significantly increase the risk of falls during activities of daily living. Addressing both cognitive and motor domains through integrated interventions, such as VR-based dual-task training, may therefore represent a promising strategy to enhance functional independence and reduce fall risk in this population. Importantly, while neurophysiological and behavioral data (e.g., EEG, eye-tracking, gait metrics) can be collected without VR, their interpretation gains depth when situated within interactive, goal-oriented VR tasks. This positions VR not merely as a stimulating modality, but as a comprehensive, data-rich ecosystem that supports both intervention and evaluation, enabling closed-loop, adaptive neurorehabilitation. Recent studies leveraging machine learning have demonstrated that the contextualization of such data enhances diagnostic precision and personalization of interventions (Rutkowski et al., 2023; Wolf et al., 2023; Alahmadi et al., 2024). Unlike traditional platforms, VR allows for the simultaneous capture and contextualization of behavioral and physiological data during dynamic task engagement, potentially uncovering patterns that are invisible in static or laboratory-based assessments. These findings are in line with findings by Tieri et al. (2018), who highlighted that VR ability to integrate movement and cognition within interactive environments leads to superior functional improvements compared to traditional paper-based or computer-based exercises. Indeed, VR environments, by eliciting real-time responses, engage sensorimotor networks that are largely ignored in conventional cognitive training. This targeted multisensory stimulation can promote neuroplastic changes, presumably offering benefits in the early stages of cognitive decline. Riva et al. (2020) further argued that VR can enhance neuroplasticity by immersing individuals in ecologically valid scenarios that simulate real-life cognitive challenges, a mechanism that was also supported by the studies included in our review, particularly those evaluating VR-based training in daily activities.

A further noteworthy result is the high level of adherence and engagement reported in VR-based training for MCI participants. VR interventions were consistently rated as engaging, motivating, and enjoyable across the studies included in this review. This supports previous literature, where Fusco and Tieri (2022) and Drigas and Sideraki (2024) highlighted that the interactive nature of VR, combined with personalized feedback, significantly improves patient adherence to rehabilitation protocols. Furthermore, Riva et al. (2020) suggested that VR promotes a greater sense of presence and immersion, which may drive motivation and increase the likelihood of sustained cognitive benefits. However, while adherence rates were high, our review also identified potential usability challenges, particularly in older adults who are unfamiliar with digital technologies. Tuena et al. (2024) found that although VR interventions were generally well accepted, some participants required assistance with navigation, calibration, and interaction with virtual environments. These findings are consistent with previous studies by Baragash et al. (2022), who indicated that technology adaptation and task simplification are essential to optimize VR usability for older adult populations. Furthermore, our review reinforces the idea that VR can improve patient engagement and motivation, a theme consistently reported in the literature. However, while VR offers clear advantages over traditional rehabilitation approaches, our results, consistent with those of Tieri et al. (2018), Riva et al. (2020), and Maggio et al. (2023) highlighted the importance of personalization, usability optimization, and strategies to mitigate cybersickness and cognitive overload in older adults. Nonetheless, translating VR-based interventions into routine clinical practice requires addressing real-world barriers, including the availability of trained personnel, cost of equipment, and digital literacy of older adults. More feasible adoption may occur within longevity clinics or specialized neurorehabilitation units, where resources and workflows allow for more innovative and individualized care models (Wilding et al., 2024). Overall, these findings support the emerging view that VR should not be conceived as a stand-alone solution, but as a hybrid platform, technologically rich, clinically adaptable, and capable of evolving into a mainstay of precision neurorehabilitation in older populations.

Finally, compared to MCI, VR-based interventions for SCD remain significantly underexplored, despite growing interest in early cognitive interventions. Our review included only one study explicitly targeting individuals with SCD, which focused on VR usability and engagement rather than cognitive outcomes. This reflects the broader gap in the literature, where very early cognitive decline is often overlooked, with research primarily focusing on interventions for diagnosed neurological conditions. However, as SCD is increasingly recognized as a precursor to MCI and dementia, future research should focus on whether early VR-based cognitive training can effectively delay or mitigate cognitive decline in at-risk populations. Arlati et al. (2021) and Baldimtsi et al. (2023) demonstrated that immersive VR interventions are feasible, well-tolerated, and engaging in individuals with SCD, with some preliminary evidence of improvements in spatial navigation and executive function. Nonetheless, more longitudinal and adequately powered studies are needed to determine their long-term efficacy on cognitive trajectories in this population.

## 5 Strengths and limitations of the study

This systematic review has several strengths that contribute to a comprehensive and in-depth assessment of VR interventions in MCI and SCD, as a prodromal phase. A major strength of this review is its rigorous methodology and adherence to the PRISMA guidelines, which ensure transparent study selection, unbiased data extraction, and structured synthesis of findings. By incorporating multiple study designs, including RCTs, non-RCTs, and feasibility assessments, this review provides a broader perspective on VR interventions. This is particularly relevant in neurorehabilitation, where usability and adherence to interventions are as crucial as clinical efficacy, especially in populations experiencing cognitive decline.

An important distinguishing feature of this review is its specific focus on both MCI and SCD populations, explicitly considering SCD as a critical early stage of cognitive decline where preventive strategies may be most effective. Unlike prior meta-analyses predominantly focused on MCI and dementia, this review emphasizes feasibility, usability, and the integration of cognitive and physical tasks within VR interventions, offering new clinical insights relevant for early-stage intervention. Furthermore, the inclusion of studies published between 2019 and 2024 ensures an up-to-date synthesis of the most recent evidence.

Another significant strength of this review is that it identifies critical gaps in the literature, particularly the limited number of studies targeting SCD and the lack of long-term follow-up. By identifying these research gaps, the review offers important directions for clinical practice and highlights priorities for future preventive interventions. Furthermore, the review addresses the need for standardized protocols and better tailoring of VR activities for individuals with cognitive impairment, emphasizing translational relevance for real-world application.

Despite these strengths, this review also has several limitations.

To critically appraise the strength of the evidence, we performed a GRADE assessment. For studies involving participants with MCI, the overall certainty of evidence was rated as low, primarily due to concerns regarding inconsistency and imprecision. Despite a low risk of bias across most studies, variability in intervention protocols and outcome measures, coupled with small sample sizes, limited the strength of conclusions. For SCD, the overall certainty was rated as low to moderate, reflecting the feasibility and usability focus of the included studies, with limited generalizability to cognitive efficacy. These ratings highlight that while VR-based interventions appear promising, current evidence remains preliminary and should be interpreted with caution. Future research employing standardized methodologies and larger samples is needed to strengthen the evidence base. Indeed, a major limitation is the heterogeneity of the included studies, particularly in intervention protocols, types of VR systems, outcome measures, and follow-up periods. The lack of standardized methodologies across studies limited our ability to directly compare interventions or conduct a meta-analysis. This reflects a broader issue within the current literature on VR interventions, rather than a limitation specific to this review. Additionally, while we included studies with both cognitive and motor training components, there was substantial variability in task design, exposure time, and assessment tools, making it difficult to determine which specific aspects of VR training contributed most to cognitive and functional improvements. Moreover, the variability in the definition of MCI across the included studies created challenges in the interpretation and comparison of findings. In most cases, MCI was conceptualized as an early stage of dementia in older adults, with little consideration for its potential heterogeneity across different neurological conditions. Only one study explicitly addressed MCI in Parkinson’s disease, but even in this case, the criteria were not clearly defined. The lack of a unified definition raises concerns about the generalizability of conclusions and represents an important area for methodological improvement in future research. To address the heterogeneity of the included studies, we opted for a structured synthesis based on target populations (e.g., MCI, SCD, mixed samples) and outcome domains (e.g., cognitive, motor, usability). While this approach does not allow for quantitative aggregation of results, it provides a narrative framework for interpreting the effects of different VR protocols. Future meta-analyses may explore moderators such as immersion level, training duration, or task complexity to identify which intervention features yield the most benefit.

While VR interventions have primarily been studied in MCI populations, their application in SCD remains largely unexplored. Given that SCD may represent an early indicator of neurodegeneration in a subset of individuals, VR-based cognitive training could serve as a preventive strategy, enhancing cognitive resilience before measurable deficits emerge. Our findings underscore the urgent need for specifically designed trials in SCD populations to establish the feasibility, effectiveness, and clinical relevance of VR interventions at this very early stage of cognitive decline.

Another limitation is that most studies did not include long-term follow-up assessments, making it unclear whether the cognitive and functional benefits of VR training persist over time. To date, only one study (Sasaninezhad et al., 2024) reported outcomes at a 3-month follow-up, but its small sample size limits the generalizability of these findings. This is a common problem in VR research, as highlighted by previous reviews (Zhu et al., 2024; Choi and Twamley, 2013; Fusco and Tieri, 2022) and highlights the need for longitudinal studies to assess the duration and stability of VR-induced cognitive benefits over time.

## 6 Future prospectives

VR holds significant potential as an innovative tool for cognitive rehabilitation in individuals with MCI and SCD (Buele et al., 2024; Choi and Lee, 2019; De Simone et al., 2023; Goumopoulos et al., 2023; Kwan et al., 2024; Liao et al., 2019; Manenti et al., 2020; Park, 2024; Sasaninezhad et al., 2024; Torpil et al., 2021; Yang et al., 2022; Zheng et al., 2025; Liao et al., 2020; Arlati et al., 2021). Its ability to simulate real-world environments in an immersive and interactive manner could enhance cognitive engagement, facilitate neuroplasticity, and improve adherence compared to traditional interventions (Maggio et al., 2023). VR-based cognitive training may be particularly beneficial in strengthening executive functions, memory retention, and cognitive-motor coordination, which are critical in delaying functional decline (Buele et al., 2024; Choi and Lee, 2019; De Simone et al., 2023; Goumopoulos et al., 2023; Kwan et al., 2024; Liao et al., 2019; Manenti et al., 2020; Park, 2024; Sasaninezhad et al., 2024; Torpil et al., 2021; Yang et al., 2022; Zheng et al., 2025; Liao et al., 2020). Additionally, dual-task VR paradigms could offer a unique advantage in training multitasking abilities and compensatory cognitive strategies, with potential applications in early dementia prevention (Brugada-Ramentol et al., 2022). Beyond cognitive enhancement, VR could serve as a diagnostic and prognostic tool, allowing for the assessment of subtle impairments in spatial navigation, attention, and executive function, which are often the earliest markers of neurodegeneration (Brugada-Ramentol et al., 2022). The integration of biomarkers such as EEG and fMRI with VR-based assessments could further refine early detection and intervention strategies.

As emphasized in this systematic review, significant gaps remain in the current body of research, particularly regarding the limited number of VR-based interventions specifically targeting SCD, the lack of long-term follow-up assessments, and the absence of biomarker integration. Future research should explore personalized VR interventions by adapting difficulty levels based on the performance of MCI and SCD patients and integrating real-time neurofeedback to optimize therapeutic outcomes. In particular, preventive strategies for SCD populations should be prioritized, given the potential for early cognitive training to delay or mitigate neurodegenerative processes. Given the current lack of studies on VR-based interventions in SCD, future research should prioritize assessing their efficacy and long-term benefits, particularly in home-based settings in which early, preventive cognitive training could be most impactful. Expanding home-based VR solutions and incorporating tele-rehabilitation models could enhance accessibility and ensure continuity of care. Finally, standardizing VR protocols and validating their efficacy through large-scale clinical trials will be essential for establishing VR as a mainstream approach in cognitive rehabilitation and dementia prevention.

Notably, one area that has not been extensively addressed in the studies included in this review but is gaining attention in the broader literature is the integration of neurophysiological markers into VR-based cognitive training. Recent studies (Drigas and Sideraki, 2024; Baragash et al., 2022) discussed the potential of using EEG-based neurofeedback in VR rehabilitation, allowing for real-time monitoring of brain activity and personalized training adjustments. Although none of the studies included in our review incorporated neurophysiological markers, future research should explore whether VR-based cognitive training can produce measurable changes in brain function, thereby supporting its role in enhancing neuroplasticity and cognitive reserve.

However, while the review highlights the high adherence rates and levels of engagement of VR interventions, usability challenges persist in older adults and individuals with cognitive impairments. Moreover, while many of the reviewed studies reported promising feasibility and adherence to VR-based interventions, these results were mostly obtained in highly structured clinical or research environments. Participants were often pre-selected based on their ability to tolerate and engage with the technology, benefiting from close supervision and technical support. As such, these findings may not fully translate to real-world conditions, where many individuals with cognitive decline face challenges such as limited digital literacy, physical or sensory impairments, and socioeconomic barriers. Moreover, while most of the VR interventions included in this review were delivered in seated or low mobility settings and are therefore compatible with the use of assistive devices such as wheelchairs, few studies explicitly reported on participants with severe mobility limitations or advanced frailty. Importantly, the feasibility of VR is highly dependent on the type of system used: non-immersive or semi-immersive platforms are often specifically designed to accommodate users with reduced mobility. Nonetheless, future studies should more systematically assess accessibility and usability in populations with diverse physical capabilities, ensuring that adaptive solutions are in place for those with significant motor impairments. To advance the clinical utility and equity of VR interventions, future trials should prioritize ecological designs that assess usability and engagement in home-based or minimally supervised settings. Integration with tele-rehabilitation platforms, simplified user interfaces, and tailored onboarding protocols could facilitate broader access and sustained adherence among diverse and at-risk populations. Addressing these usability issues is crucial to ensure that VR interventions are accessible, acceptable, and scalable for real-world clinical application in vulnerable populations. These efforts will help ensure that VR-based cognitive training is not only effective in principle but also grounded in the embodied realities of aging.

In addition, while the review refers to cognitive benefits across the included studies, few works explicitly delineate the theoretical frameworks or cognitive mechanisms underlying these outcomes. VR-based interventions often involve tasks that stimulate domains such as attention, working memory, executive functions, and spatial navigation through goal-oriented, multisensory, and interactive activities. However, the extent to which specific cognitive processes are targeted remains inconsistently reported. Future research should more clearly define the cognitive constructs being trained, align intervention designs with established cognitive models, and employ standardized neuropsychological measures to assess domain-specific effects. Clarifying these mechanisms will help establish the cognitive specificity and clinical relevance of VR-based training protocols.

Finally, future research should incorporate critical perspectives from the field of gerontechnology. As highlighted by Peine et al. (2015) and Peine and Neven (2021) the widely promoted “triple-win narrative,” which assumes equal benefits for technology developers, policymakers, and older users, often overlooks the practical, economic, and socio-cultural barriers that hinder widespread adoption in real-world contexts. A more nuanced understanding of these dynamics could help move beyond a simplistic technology-acceptance model and support the development, implementation, and dissemination of VR interventions that are both accessible and sustainable for older adults.

## 7 Conclusion

This systematic review provides relevant evidence that VR-based interventions could be effective for cognitive rehabilitation in individuals with early cognitive impairment. The major evidence was observed for MCI, where VR interventions could improve executive function, memory, and cognitive-motor performance. In the few studies of SCD, VR was well tolerated and engaging, although further research is needed to assess its long-term impact on preventing cognitive decline. In conclusion, VR represents a promising tool for cognitive rehabilitation in individuals with MCI and SCD, but further refinement of intervention protocols, adaptation to different cognitive stages, and integration into clinical practice are needed to maximize its potential benefits.

## Data Availability

The original contributions presented in the study are included in the article/Supplementary material, further inquiries can be directed to the corresponding author.

## References

[ref8001] Adamson M. M. Main K. Harris O. A. Kang X. (2021). Sex differences in cortical thickness and diffusion properties in patients with traumatic brain injury: a pilot study. Brain Inj. 36, 488–502. 10.1080/02699052.2022.2034046 35113752

[ref1] Alahmadi T. J. Rahman A. U. Alhababi Z. A. Ali S. Alkahtani H. K. (2024). Prediction of mild cognitive impairment using EEG signal and BiLSTM network. Mach. Learn. Sci. Technol. 5:025028. doi: 10.1088/2632-2153/ad38fe

[ref2] Arlati S. Di Santo S. G. Franchini F. Mondellini M. Filiputti B. Luchi M. . (2021). Acceptance and usability of immersive virtual reality in older adults with objective and subjective cognitive decline. J. Alzheimers Dis. JAD. 80, 1025–1038. doi: 10.3233/JAD-201431, PMID: 33646164

[ref3] Baldimtsi E. Mouzakidis C. Karathanasi E. M. Verykouki E. Hassandra M. Galanis E. . (2023). Effects of virtual reality physical and cognitive training intervention on cognitive abilities of elders with mild cognitive impairment. J. Alzheimers Dis. Rep. 7, 1475–1490. doi: 10.3233/ADR-230099, PMID: 38225966 PMC10789285

[ref4] Baragash R. S. Aldowah H. Ghazal S. (2022). Virtual and augmented reality applications to improve older adults’ quality of life: a systematic mapping review and future directions. Digit. Health 8:20552076221132099. doi: 10.1177/20552076221132099, PMID: 36339904 PMC9629585

[ref5] Bassett S. S. Folstein M. F. (1993). Memory complaint, memory performance, and psychiatric diagnosis: a community study. J. Geriatr. Psychiatry Neurol. 6, 105–111. doi: 10.1177/089198879300600207, PMID: 8512626

[ref6] Bessi V. Mazzeo S. Padiglioni S. Piccini C. Nacmias B. Sorbi S. . (2018). From subjective cognitive decline to Alzheimer’s disease: the predictive role of neuropsychological assessment, personality traits, and cognitive reserve. A 7-year follow-up study. J Alzheimer's Dis 63, 1523–1535. doi: 10.3233/JAD-171180, PMID: 29782316

[ref7] Brugada-Ramentol V. Bozorgzadeh A. Jalali H. (2022). Enhance VR: a multisensory approach to cognitive training and monitoring. Front Digit Health. 4:916052. doi: 10.3389/fdgth.2022.916052, PMID: 35721794 PMC9203823

[ref8] Buele J. Avilés-Castillo F. Del-Valle-Soto C. Varela-Aldás J. Palacios-Navarro G. (2024). Effects of a dual intervention (motor and virtual reality-based cognitive) on cognition in patients with mild cognitive impairment: a single-blind, randomized controlled trial. J. NeuroEng. Rehabil. 21:130. doi: 10.1186/s12984-024-01422-w, PMID: 39090664 PMC11293003

[ref9] Cabinio M. Rossetto F. Isernia S. Saibene F. L. Di Cesare M. Borgnis F. . (2020). The use of a virtual reality platform for the assessment of the memory decline and the hippocampal neural injury in subjects with mild cognitive impairment: the validity of Smart aging serious game (SASG). J. Clin. Med. 9:1355. doi: 10.3390/jcm9051355, PMID: 32384591 PMC7290592

[ref10] Campbell M. McKenzie J. E. Sowden A. Katikireddi S. V. Brennan S. E. Ellis S. . (2020). Synthesis without meta-analysis (SWiM) in systematic reviews: reporting guideline. BMJ 368:l6890. doi: 10.1136/bmj.l6890, PMID: 31948937 PMC7190266

[ref11] Chiaramonte R. Cioni M. (2021). Critical spatiotemporal gait parameters for individuals with dementia: a systematic review and meta-analysis. Hong Kong Physiother. J. 41, 1–14. doi: 10.1142/S101370252130001X, PMID: 34054252 PMC8158408

[ref12] Choi W. Lee S. (2019). The effects of virtual kayak paddling exercise on postural balance, muscle performance, and cognitive function in older adults with mild cognitive impairment: a randomized controlled trial. J. Aging Phys. Act. 27, 861–870. doi: 10.1123/japa.2018-0020, PMID: 31034303

[ref13] Choi J. Twamley E. W. (2013). Cognitive rehabilitation therapies for Alzheimer’s disease: a review of methods to improve treatment engagement and self-efficacy. Neuropsychol. Rev. 23, 48–62. doi: 10.1007/s11065-013-9227-4, PMID: 23400790 PMC3596462

[ref14] De Luca R. Gangemi A. Maggio M. G. Bonanno M. Calderone A. Mazzurco Masi V. M. . (2024). Effects of virtual rehabilitation training on post-stroke executive and praxis skills and depression symptoms: a quasi-randomised clinical trial. Diagn. Basel Switz. 14:1892. doi: 10.3390/diagnostics14171892 PMC11394403 39272676

[ref15] De Simone M. S. Costa A. Tieri G. Taglieri S. Cona G. Fiorenzato E. . (2023). The effectiveness of an immersive virtual reality and telemedicine-based cognitive intervention on prospective memory in Parkinson’s disease patients with mild cognitive impairment and healthy aged individuals: design and preliminary baseline results of a placebo-controlled study. Front. Psychol. 14:1268337. doi: 10.3389/fpsyg.2023.1268337 37928597 PMC10622796

[ref16] Drigas A. Sideraki A. (2024). Brain neuroplasticity leveraging virtual reality and brain-computer Interface technologies. Sensors 24:5725. doi: 10.3390/s24175725, PMID: 39275636 PMC11397861

[ref17] Duff K. (2024). Mild cognitive impairment. Neurol. Clin. 42, 781–792. doi: 10.1016/j.ncl.2024.05.007, PMID: 39343474

[ref18] Fusco A. Tieri G. (2022). Challenges and perspectives for clinical applications of immersive and non-immersive virtual reality. J. Clin. Med. 11:4540. doi: 10.3390/jcm11154540, PMID: 35956157 PMC9369665

[ref19] GjØra L. Strand B. H. Bergh S. Borza T. Brækhus A. Engedal K. . (2021). Current and future prevalence estimates of mild cognitive impairment, dementia, and its subtypes in a population-based sample of people 70 years and older in Norway: the HUNT study. J Alzheimer's Dis 79, 1213–1226. doi: 10.3233/JAD-201275, PMID: 33427745 PMC7990439

[ref20] Goumopoulos C. Skikos G. Frounta M. (2023). Feasibility and effects of cognitive training with the COGNIPLAT game platform in elderly with mild cognitive impairment: pilot randomized controlled trial. Games Health J. 12, 414–425. doi: 10.1089/g4h.2023.0029, PMID: 37276027

[ref21] Guyatt G. H. Oxman A. D. Vist G. E. Kunz R. Falck-Ytter Y. Alonso-Coello P. . (2008). GRADE: an emerging consensus on rating quality of evidence and strength of recommendations. BMJ 336, 924–926. doi: 10.1136/bmj.39489.470347.AD, PMID: 18436948 PMC2335261

[ref22] Haddaway N. R. Page M. J. Pritchard C. C. McGuinness L. A. (2022). PRISMA2020: an R package and shiny app for producing PRISMA 2020-compliant flow diagrams, with interactivity for optimised digital transparency and open synthesis. Campbell Syst. Rev. 18:e1230. doi: 10.1002/cl2.1230, PMID: 36911350 PMC8958186

[ref23] Jack C. R. Albert M. Knopman D. S. McKhann G. M. Sperling R. A. Carillo M. . (2011). Introduction to revised criteria for the diagnosis of Alzheimer’s disease: national institute on aging and the Alzheimer association workgroups. Alzheimers Dement. J. Alzheimers Assoc. 7, 257–262. doi: 10.1016/j.jalz.2011.03.004 PMC3096735 21514247

[ref24] Jessen F. Amariglio R. E. Buckley R. F. van der Flier W. M. Han Y. Molinuevo J. L. . (2020). The characterisation of subjective cognitive decline. Lancet Neurol. 19, 271–278. doi: 10.1016/S1474-4422(19)30368-0, PMID: 31958406 PMC7062546

[ref25] Jessen F. Amariglio R. E. van Boxtel M. Breteler M. Ceccaldi M. Chételat G. . (2014). A conceptual framework for research on subjective cognitive decline in preclinical Alzheimer’s disease. Alzheimers Dement. 10, 844–852. doi: 10.1016/j.jalz.2014.01.001, PMID: 24798886 PMC4317324

[ref26] Kim O. Pang Y. Kim J. H. (2019). The effectiveness of virtual reality for people with mild cognitive impairment or dementia: a meta-analysis. BMC Psychiatry 19:219. doi: 10.1186/s12888-019-2180-x, PMID: 31299921 PMC6626425

[ref27] Kwan R. Y. C. Liu J. Sin O. S. K. Fong K. N. K. Qin J. Wong J. C. Y. . (2024). Effects of virtual reality motor-cognitive training for older people with cognitive frailty: Multicentered randomized controlled trial. J. Med. Internet Res. 26:e57809. doi: 10.2196/57809, PMID: 39259959 PMC11425022

[ref28] Latella D. Formica C. Ielo A. Grioli P. Marra A. Costanzo D. . (2024). A feasibility and usability study of a virtual reality tool (VESPA 2.0) for cognitive rehabilitation in patients with mild cognitive impairment: an ecological approach. Front. Psychol. 15:1402894. doi: 10.3389/fpsyg.2024.1402894, PMID: 39492810 PMC11529225

[ref9001] Levy A. M. Saling M. M. Anderson J. F. I. (2023). Psychological distress and gender predict cognitive complaint after adult civilian mild traumatic brain injury in pre-morbidly healthy adults. Neuropsychol. Rehabil. 34, 721–741. doi: 10.1080/09602011.2023.2236348 37493086

[ref29] Liao Y. Y. Chen I. H. Lin Y. J. Chen Y. Hsu W. C. (2019). Effects of virtual reality-based physical and cognitive training on executive function and dual-task gait performance in older adults with mild cognitive impairment: a randomized control trial. Front. Aging Neurosci. 11:162. doi: 10.3389/fnagi.2019.00162 31379553 PMC6646677

[ref30] Liao Y. Y. Tseng H. Y. Lin Y. J. Wang C. J. Hsu W. C. (2020). Using virtual reality-based training to improve cognitive function, instrumental activities of daily living and neural efficiency in older adults with mild cognitive impairment. Eur. J. Phys. Rehabil. Med. 56, 47–57. doi: 10.23736/S1973-9087.19.05899-4, PMID: 31615196

[ref31] Liu Q. Chen B. Wang Q. Xu D. Yang M. Lin G. . (2025). Sex differences in the relationship between olfactory and cognitive impairment among subjects with subjective cognitive decline and mild cognitive impairment. Biol. Sex Differ. 16:12. doi: 10.1186/s13293-025-00691-x, PMID: 39948615 PMC11827212

[ref32] Maggio M. G. Baglio F. Arcuri F. Borgnis F. Contrada M. Diaz M. D. M. . (2024). Cognitive telerehabilitation: an expert consensus paper on current evidence and future perspective. Front. Neurol. 15:1338873. doi: 10.3389/fneur.2024.1338873, PMID: 38426164 PMC10902044

[ref33] Maggio M. G. Cezar R. P. Milardi D. Borzelli D. DE Marchis C. D’Avella A. . (2023). Do patients with neurological disorders benefit from immersive virtual reality? A scoping review on the emerging use of the computer-assisted rehabilitation environment. Eur. J. Phys. Rehabil. Med. 60, 37–43. doi: 10.23736/S1973-9087.23.08025-5 37971719 PMC10939039

[ref34] Maggio M. G. Piazzitta D. Andaloro A. Latella D. Sciarrone F. Casella C. . (2022). Embodied cognition in neurodegenerative disorders: what do we know so far? A narrative review focusing on the mirror neuron system and clinical applications. J. Clin. Neurosci. 98, 66–72. doi: 10.1016/j.jocn.2022.01.028, PMID: 35134659

[ref35] Manenti R. Gobbi E. Baglio F. Macis A. Ferrari C. Pagnoni I. . (2020). Effectiveness of an innovative cognitive treatment and telerehabilitation on subjects with mild cognitive impairment: a multicenter, randomized, active-controlled study. Front. Aging Neurosci. 12:585988. doi: 10.3389/fnagi.2020.585988, PMID: 33304267 PMC7701275

[ref36] Morone G. Tramontano M. Iosa M. Shofany J. Iemma A. Musicco M. . (2014). The efficacy of balance training with video game-based therapy in subacute stroke patients: a randomized controlled trial. Biomed. Res. Int. 2014:580861. doi: 10.1155/2014/580861, PMID: 24877116 PMC4026958

[ref9002] Neumann D. Sander A. M. Perkins S. M. Bhamidipalli S. S. Hammond F. M. (2021). Negative attribution bias and related risk factors after brain injury. J. Head Trauma Rehabil. 36, E61–E70. 32769831 10.1097/HTR.0000000000000600 PMC7769858

[ref37] Ouzzani M. Hammady H. Fedorowicz Z. Elmagarmid A. (2016). Rayyan-a web and mobile app for systematic reviews. Syst. Rev. 5:210. doi: 10.1186/s13643-016-0384-4, PMID: 27919275 PMC5139140

[ref38] Page M. J. McKenzie J. E. Bossuyt P. M. Boutron I. Hoffmann T. C. Mulrow C. D. . (2021). The PRISMA 2020 statement: an updated guideline for reporting systematic reviews. BMJ:n71. doi: 10.1136/bmj.n71 33782057 PMC8005924

[ref39] Park J. H. (2024). Is virtual reality-based cognitive training in parallel with functional near-infrared spectroscopy-derived neurofeedback beneficial to improve cognitive function in older adults with mild cognitive impairment? Disabil. Rehabil. 47, 1717–1724. doi: 10.1080/09638288.2024.2380483 39033386

[ref40] Peine A. Faulkner A. Jæger B. Moors E. (2015). Science, technology and the ‘grand challenge’ of ageing—understanding the socio-material constitution of later life. Technol. Forecast. Soc. Change 93, 1–9. doi: 10.1016/j.techfore.2014.11.010

[ref41] Peine A. Neven L. (2021). The co-constitution of ageing and technology – a model and agenda. Ageing Soc. 41, 2845–2866. doi: 10.1017/S0144686X20000641

[ref43] Rabin L. A. Smart C. M. Amariglio R. E. (2017). Subjective cognitive decline in preclinical Alzheimer’s disease. Annu. Rev. Clin. Psychol. 13, 369–396. doi: 10.1146/annurev-clinpsy-032816-045136, PMID: 28482688

[ref44] Rabin L. A. Smart C. M. Crane P. K. Amariglio R. E. Berman L. M. Boada M. . (2015). Subjective cognitive decline in older adults: an overview of self-report measures used across 19 international research studies. J Alzheimer's Dis 48, S63–S86. doi: 10.3233/JAD-150154 26402085 PMC4617342

[ref45] Reisberg B. Shulman M. B. Torossian C. Leng L. Zhu W. (2010). Outcome over seven years of healthy adults with and without subjective cognitive impairment. Alzheimers Dement. 6, 11–24. doi: 10.1016/j.jalz.2009.10.002, PMID: 20129317 PMC3873197

[ref46] Riva G. Mancuso V. Cavedoni S. Stramba-Badiale C. (2020). Virtual reality in neurorehabilitation: a review of its effects on multiple cognitive domains. Expert Rev. Med. Devices 17, 1035–1061. doi: 10.1080/17434440.2020.1825939, PMID: 32962433

[ref47] Rutkowski T. M. Abe M. S. Komendzinski T. Sugimoto H. Narebski S. Otake-Matsuura M. (2023). Machine learning approach for early onset dementia neurobiomarker using EEG network topology features. Front. Hum. Neurosci. 17:1155194. doi: 10.3389/fnhum.2023.1155194 37397858 PMC10311997

[ref48] Sachdev P. S. Blacker D. Blazer D. G. Ganguli M. Jeste D. V. Paulsen J. S. . (2014). Classifying neurocognitive disorders: the DSM-5 approach. Nat. Rev. Neurol. 10, 634–642. doi: 10.1038/nrneurol.2014.181, PMID: 25266297

[ref49] Sasaninezhad M. Moradi A. Farahimanesh S. Choobin M. H. Almasi-Dooghaee M. (2024). Enhancing cognitive flexibility and working memory in individuals with mild cognitive impairment: exploring the impact of virtual reality on daily life activities. Geriatr. Nurs. 56, 32–39. doi: 10.1016/j.gerinurse.2023.12.008, PMID: 38211369

[ref50] Specht J. Stegmann B. Gross H. Krakow K. (2023). Cognitive training with head-mounted display virtual reality in neurorehabilitation: pilot randomized controlled trial. JMIR Serious Games 11:e45816. doi: 10.2196/45816, PMID: 37477957 PMC10403796

[ref9003] Stafslien E. D. Turkstra L. S. (2020). Sex-based differences in expectations for social communication after TBI. Brain injury. 34, 1756–1776. 33222531 10.1080/02699052.2020.1849799

[ref51] Sterne J. A. Hernán M. A. Reeves B. C. Savović J. Berkman N. D. Viswanathan M. . (2016). ROBINS-I: a tool for assessing risk of bias in non-randomised studies of interventions. BMJ 355:i4919. doi: 10.1136/bmj.i4919 27733354 PMC5062054

[ref52] Sterne J. A. C. Savović J. Page M. J. Elbers R. G. Blencowe N. S. Boutron I. . (2019). RoB 2: a revised tool for assessing risk of bias in randomised trials. BMJ 366:l4898. doi: 10.1136/bmj.l4898, PMID: 31462531

[ref9004] Teterina A. Zulbayar S. Mollayeva T. Chan V. Colantonio A. Escobar M. (2023). Gender versus sex in predicting outcomes of traumatic brain injury: a cohort study utilizing large administrative databases. Sci. Rep. 13:18453. doi: 10.1038/s41598-023-45683-2 37891419 PMC10611793

[ref53] Tieri G. Morone G. Paolucci S. Iosa M. (2018). Virtual reality in cognitive and motor rehabilitation: facts, fiction and fallacies. Expert Rev. Med. Devices 15, 107–117. doi: 10.1080/17434440.2018.1425613, PMID: 29313388

[ref54] Torpil B. Şahin S. Pekçetin S. Uyanık M. (2021). The effectiveness of a virtual reality-based intervention on cognitive functions in older adults with mild cognitive impairment: a single-blind, randomized controlled trial. Games Health J. 10, 109–114. doi: 10.1089/g4h.2020.0086, PMID: 33058735

[ref55] Tuena C. Serino S. Goulene K. M. Pedroli E. Stramba-Badiale M. Riva G. (2024). Bodily and visual-cognitive navigation aids to enhance spatial recall in mild cognitive impairment. J Alzheimer's Dis 99, 899–910. doi: 10.3233/JAD-240122, PMID: 38701150 PMC11191438

[ref56] Tuokko H. A. Smart C. M. (2018). Neuropsychology of cognitive decline: a developmental approach to assessment and intervention. New York, NY, USA: Guilford Publications.

[ref57] Voinescu A. Papaioannou T. Petrini K. Stanton Fraser D. (2024). Exergaming for dementia and mild cognitive impairment. Cochrane Database Syst. Rev. 2024:CD013853. doi: 10.1002/14651858.CD013853.pub2 PMC11423707 39319863

[ref8002] Wågberg S. Stålnacke B. M. Magnusson B. M. (2023). Gender and age differences in outcomes after mild traumatic brain injury. J. Clin. Med. 12:4883. 37568285 10.3390/jcm12154883 PMC10419972

[ref58] Wilding R. Barbosa Neves B. Waycott J. Miller E. Porter T. Johnston J. . (2024). Introducing virtual reality to older adults: a qualitative analysis of a co-design innovation with care staff. Arch. Gerontol. Geriatr. 125:105505. doi: 10.1016/j.archger.2024.105505, PMID: 38851090

[ref59] Wion R. K. Hill N. L. DePasquale N. Mogle J. Whitaker E. B. (2020). The relationship between subjective cognitive impairment and activity participation: a systematic review. Acta Adapt. Aging 44, 225–245. doi: 10.1080/01924788.2019.1651188 PMC8009485 33790489

[ref60] Wolf A. Tripanpitak K. Umeda S. Otake-Matsuura M. (2023). Eye-tracking paradigms for the assessment of mild cognitive impairment: a systematic review. Front. Psychol. 14:1197567. doi: 10.3389/fpsyg.2023.1197567 37546488 PMC10399700

[ref9005] World Health Organization (WHO). (2021). Classification of Tumours Editorial Board. Central Nervous System Tumours: WHO Classification of Tumours. 5th ed. Vol 6. International Agency for Research on Cancer (IARC).

[ref61] Yang J. G. Thapa N. Park H. J. Bae S. Park K. W. Park J. H. . (2022). Virtual reality and exercise training enhance brain, cognitive, and physical health in older adults with mild cognitive impairment. Int. J. Environ. Res. Public Health 19:13300. doi: 10.3390/ijerph192013300, PMID: 36293881 PMC9602597

[ref62] Yang Q. Zhang L. Chang F. Yang H. Chen B. Liu Z. (2025). Virtual reality interventions for older adults with mild cognitive impairment: systematic review and Meta-analysis of randomized controlled trials. J. Med. Internet Res. 27:e59195. doi: 10.2196/59195, PMID: 39793970 PMC11759915

[ref63] Zheng L. Li X. Xu Y. Yang Y. Wan X. Ma X. . (2025). Effects of virtual reality-based activities of daily living rehabilitation training in older adults with cognitive frailty and activities of daily living impairments: a randomized controlled trial. J. Am. Med. Dir. Assoc. 26:105397. doi: 10.1016/j.jamda.2024.105397, PMID: 39615543

[ref64] Zhu C. Io M. U. Hall C. M. Ngan H. F. B. Peralta R. L. (2024). How to use augmented reality to promote a destination? The mediating role of augmented reality attachment. Int. J. Tour. Res. 26:e2603. doi: 10.1002/jtr.2603

