# Supplementary material for: Cognitive rehabilitation using virtual reality in subjective cognitive decline and mild cognitive impairment: a systematic review
Source: Front Psychol. 2025 Sep 9;16:1641693. doi: 10.3389/fpsyg.2025.1641693 (PMC12454054; doi:10.3389/fpsyg.2025.1641693)
Supplement: Supplementary file 1 [file Table_1.DOCX]

# Supplementary table 1. Summary Table of VR Interventions and Outcomes

| **Study** | **Type of VR** | **Positive Outcomes** | **Negative Outcomes** |
| --- | --- | --- | --- |
| Sasaninezhad et al., 2024 | Non-immersive VR | Improvements were registered in global working memory (follow-up: EG= 7.20; CG= 5.10), daily functioning (follow-up: EG=6.30; CG:4.25), anxiety (follow-up: EG= 2.70; CG= 3.70) and depression (follow-up: EG= 2.65; CG= 4.25) | None reported |
| Zheng et al., 2025 | Fully immersive VR | Compared with the CG, the EG showed significant improvements in IADL performance (EG= 13.94; CG= 9.18), cognition (EG= 24.73; CG= 20.18), frailty (EG= 2; CG= 3) and depression (EG= 2; CG= 5) | Minor dropout (9%) |
| Liao et al., 2020 | Immersive VR | Both groups showed improved executive function and verbal memory. The EG showed significant improvements in global cognition (p<0.001), verbal memory (delayed recall, p=0.002), and IADL (p<0.001). In the EG, hemodynamic data revealed decreased activation in prefrontal areas after training (p=0.0015), indicative of increased neural efficiency | None reported |
| Manenti et al., 2020 | Semi-immersive VR + Telerehabilitation | Improvements in memory (EG= 33.4; CG= 31.5), attention (EG= 64.5; CG= 60) and language (EG= 29.8; CG= 30.4); Long-term cognitive benefits were also registered | High adherence, minor technical issues |
| De Simone et al., 2023 | Immersive VR | Improvements were registered in: executive functions measured with Stroop Test (EG= 18.5; CG= 12.9), daily-life abilities and high usability (92% of the overall sample provided rates ranging from 4 to 5 on the Likert scale) | Minor cybersickness (1 dropout) |
| Goumopoulos et al., 2023 | Serious Game + AR/VR elements | Compared with the CG, the EG showed improvements in global cognition (EG= 12.5; CG= 21.5) | High adherence, no major adverse events |
| Yang et al., 2022 | Immersive VR | Overall Cognition improvements: MMSE (VRCT group= 28.1; CG= 26.7. p<0.05) and SDST (VRCT group= 53.2; CG= 32.1. p<0.05) | None significant |
| Park, 2024 | Non-immersive VR + Neurofeedback | There was significant effect of time on the TMT-B (p < 0.001) and the BDST (p < 0.001), working memory and neural efficiency (p < 0.001) | None reported |
| Choi and Lee, 2019 | Non-immersive VR | The EG showed significant improvements of in the motor-free visual perception test-3 (MVPT-3) score (+7), in the Berg balance scale (BBS) score (+3), and in the timed up and go test (TUG) test (–1.92), with all results being significantly better than those of the CG group (p<0.05) | None reported |
| Cho Kwan et al., 2024 | Immersive VR | VRMCT was effective in promoting global cognitive function (p=.03), marginally promoting executive function (p=.07), and reducing frailty (p=.03). | Mild VR sickness (rare) |
| Liao et al., 2019 | Non-immersive VR | Improvements were reported in cognitive dual task gait performance (EG= 82.5±30.6; CG= 78.1±33.2) | None reported |
| Buele et al., 2024 | Immersive VR | Between groups comparison did not reveal significant differences in either cognitive function or geriatric depression. There was no statistically significant improvement in any of the groups when evaluating their performance in IADLs (CG, p = 0.28; EG, p = 0.46). The completion rate in the EG was higher (82.35%) compared to the CG (70.59%) | Minor dropouts, high adherence |
| Torpil et al., 2021 | Non-immersive VR | Within group analysis showed a significant increase in all functions (p<0.001) in except praxis and memory in the EG (<0.05), and in the CG (P<0.05). Between group analysis showed a significant increase in orientation, visual-spatial perception, visuo-motor organization, thinking operation, and attention/concentration in the EG | None reported |
| Baldimtsi et al., 2023 | Immersive VR | There were significant improvements in general cognition (VRADA group= -0.210; CG= -0.041), verbal memory (VRADA group=0.038; CG= 0.518), executive function (VRADA group= 16.81; CG= -20.18) | None significant |
| Cabinio et al., 2020 | Non-immersive VR Serious Game | Data reveals that for the MoCA test total score the EG (22.26±2.84) performance was within the normal range but significantly worse than the CG (26.97±2.35) | None reported |
| Tuena et al., 2024 | Immersive VR | Results showed that the bodily condition yielded better spatial memory compared to vision only (p=0.047), the interactive allocentric map significantly improved spatial memory compared to free navigation (p=0.021) and the bodily condition was superior compared to the free navigation (p=0.003) | Minor VR discomfort (1 withdrawal) |
| Latella et al., 2024 | Tablet-based VR | Significant improvements in executive functions (p = 0.005) and memory functions (p<0.001) | No significant |
| Arlati et al., 2021 | Immersive VR | Participants enjoyed the experience (spatial presence 3.51±0.50, engagement 3.85±0.68, naturalness 3.85±0.82) and reported negligible side-effects (SSQ: 3.74) | Minor cybersickness, 1 fall (no injury) |

VR: Virtual Reality
